# TCam-2 Seminoma Cells Exposed to Egg-Derived Microenvironment Modify Their Shape, Adhesive Pattern and Migratory Behaviour: A Molecular and Morphometric Analysis

**DOI:** 10.1371/journal.pone.0076192

**Published:** 2013-10-01

**Authors:** Francesca Ferranti, Fabrizio D’Anselmi, Maria Caruso, Vittorio Lei, Simona Dinicola, Alessia Pasqualato, Alessandra Cucina, Alessandro Palombo, Giulia Ricci, Angela Catizone, Mariano Bizzarri

**Affiliations:** 1 Department of Anatomy, Histology, Forensic Medicine and Orthopedics - Section of Histology and Medical Embryology, Sapienza University of Rome, Rome, Italy; 2 Department of Surgery “PietroValdoni”, Sapienza University of Rome, Rome, Italy; 3 Department of Experimental Medicine, Sapienza University of Rome, Rome, Italy; 4 Farnia srl, Rome, Italy; 5 Department of Clinical and Molecular Medicine, Sapienza University of Rome, Rome, Italy; 6 Department of Neuroscience and Imaging, Section of Physiology and Physiopathology, “G. D’Annunzio” University of Chieti-Pescara, Chieti, Italy; 7 Department of Experimental Medicine, Second University of Naples, Naples, Italy; Thomas Jefferson University, United States of America

## Abstract

Seminoma is one of the most common Testicular Germ Cell Tumours that originates during embryonic development due to an alteration of the local niche that in turn results in a delayed or blocked differentiation of Primordial Germ Cells. The block of differentiation is actually a common way to develop cancer disease as postulated by the "embryonic rest theory of cancer". In agreement with this theory different studies have demonstrated that embryonic cues display the capacity of reprogramming aggressive cancer cells towards a less aggressive phenotype. Herein we investigate the ability of a culture medium added with 10% egg albumen (EW, Egg White) to modulate seminoma cell phenotype and behaviour, by ensuring a proper set of morphogenetic signals. We chose to use the TCam-2 seminoma cell line that has been established as the only available cell line, obtained from a primary testicular seminoma. EW is able to: 1) modify TCam-2 cell spreading rate and cell-substrate adhesion without affecting proliferation and survival indexes; 2) modulate TCam-2 actin distribution pattern increasing cortical localization of actin filaments; 3) increase TCam-2 cell-cell junction capability; 4) decrease both chemo-sensitive and collective TCam-2 migratory behaviour. According to these observations morphometric fractal analysis revealed the ability of EW to increase Circularity and Solidity parameters and, consequently, to decrease Fractal dimension. Prompted by these observations we hypothesize that EW treatment could rescue, at least in part, the neoplastic-metastatic behaviour of seminoma cells.

## Introduction

Cancer stem cells share many features and behaviours with their embryonic progenitors. This observation gave rise to the ‘embryonic rest’ theory of cancer origin [1,2] which suggests that differentiated tissues contain dormant embryonic remnants that may give rise to cancer lesions when activated in the differentiated tissue microenvironments [1-4]. In this regard Testicular Germ Cell Tumours (TGCTs), are known to be the most frequent solid malignant tumours in 15 to 39 year-old men [5] and originate during embryonic development when Primordial Germ Cells (PGCs) or gonocytes are converted into a pre-invasive lesion, known as carcinoma in situ (CIS). The initiation of this neoplastic transformation is most likely triggered by a disruption of the local embryonic niche [6,7] that results in a delayed or blocked differentiation of embryonic germ cells: CIS cells share, in fact, morphological and molecular similarities with PGC and early gonocytes [6] and this lesion appears frequently in patients affected by varying degree of gonadal dysgenesis [8]. Seminomas represent more than 50% of all TGCTs and are considered one of the possible progressions of CIS lesions [9]. During the development of seminomas, CIS cells become gradually independent from the intra-testicular molecular signals, fill up the lumen of the seminiferous tubules and adopt a metastatic behaviour in the last stage of seminoma cell tumour progression.

Different experimental models demonstrated that an embryonic microenvironment might have the capacity to reverse the phenotype of cancer cells [10-16]. Moreover, in our previous studies, the culture medium filled with 10% egg albumen (EW, Egg White) was added to MCF7 cells and was demonstrated to be effective in reverting their neoplastic phenotype [17,18].

Herein we investigated the ability of EW treatment to modulate seminoma phenotype and its neoplastic behaviour, by ensuring a proper set of morphogenetic cues.

Currently, TCam-2 cell line [19-22] has been established as the only available cell line, obtained from a primary testicular seminoma of a 35-year-old patient [22].

Our study was aimed to investigate how EW could modify TCam-2 cell proliferation, survival, shape and cytoskeleton patterning, adhesive and migratory behaviour.

Experimental data presented here support the hypothesis by which embryonic cues may induce seminoma cell differentiation, generating more organized and coherent epithelial-like structures.

## Materials and Methods

### EW

The EW (Egg White) is the albumen derived from unfertilized hen’s egg, easily soluble in culture media. EW has been patented (patent number 0001400147) for its use in 2D and 3D cultures. In all described experiments EW was dissolved in the cell culture medium to reach a final concentration of 10% of the volume. The composition of EW has been reported by several authors, however, a compelling and exhaustive composition survey of the hen’s egg albumen still await to be defined, as new components have been recently identified, and others will presumably continue to be ascertained through the use of powerful new technologies [23].

### TCam-2 cell cultures

The TCam-2 human cell line was derived in 1993 from a primary testicular tumour sample of pure classical seminoma [22]. TCam-2 cells were cultured in RPMI 1640 (Lonza) supplemented with 10% Fetal Bovine Serum (FBS, Lonza) and penicillin/streptomycin (Invitrogen) at 37°C in a humidified atmosphere with 5% carbon dioxide [21]. When indicated, 10% EW was added to the culture medium. Culture span depended on the different assays performed. The time 0 plating cell density is 1.5x10^4^/cm^2^ if not differently specified in the text.

### Proliferation and survival assays

To test the effect of EW on cell proliferation and survival, TCam-2 cells were cultured in the presence or absence of 10% EW. After 24, 48 and 72 hours, cells were trypsinized, harvested and counted. Dead cells were evaluated using the trypan blue exclusion staining technique.

In order to assess the effect of EW on cell cycle entry, the cells were cultured with or without 10% EW for 24, 48 and 72 hours. The cells were collected and stained with a propidium iodide/RNase solution (50 μg⁄mL and 100 U⁄mL respectively) for at least 3 hours. The cell suspensions were analysed on a Beckman Coulter Epics XL Flow Cytometer.

### Adhesion assay

As TCam-2 adhesion assay, cells were cultured for 40 min at 37°C with or without 10% EW. Adhesion assays were performed on plastic dishes pre-coated or not with 10 µg/ml laminin (Sigma-Aldrich) or fibronectin (Sigma-Aldrich) as previously described by Di Florio et coll, 2007 [24]. Adherent cells, after suspended cell removal, were collected by trypsinization and then counted in Thoma’s chamber.

### Cell spreading and Computerized Time-Lapse Video Analysis

In order to evaluate TCam-2 cell spreading capability, in the presence or absence of 10% EW, TCam-2 cells were cultured on Ibidi microscopy chamber (Ibidi 132042) and subjected to video microscopy for 50 min at 37°C. These cells were allowed to spread on Ibidi chambers pre-coated or not with 10 µg/ml laminin (Sigma-Aldrich) or fibronectin (Sigma-Aldrich). Time-lapse video analysis was performed using a Leica Laser Scanning TCS SP2 confocal microscopy. Images were captured every 1 minute and movies were generated using the Leica confocal software. Every ten minutes spread cells of control and treated samples were counted.

### Western blot analysis

Cultured cells were homogenised in RIPA buffer (Sigma-Aldrich) and the protein contents were determined using the Bradford protein assay (Biorad Laboratories). Proteins (70 µg per lane) were re-suspended in boiling Laemmli buffer under reducing conditions. Proteins were separated on 10% or 7% SDS–PAGE gels depending on the predicted molecular weight of the investigated proteins. Gels were then electrotransferred to nitrocellulose membranes (Protran). Non-specific antibody binding was blocked by incubation with 5% no-fat milk in TBS buffer (20 mM Tris pH 7.6, 150 mM NaCl). After the blocking step, membranes were incubated in 5% no-fat milk in TBS, for 16 hours at 4°C, with the antibodies listed below: anti-E-cadherin (Santa Cruz Biotechnology, rabbit polyclonal sc-7870 1:200), anti-β-catenin (Santa Cruz Biotechnology, mouse monoclonal sc-7963, 1:200 dilution), anti-connexin-43 (Sigma-Aldrich; rabbit polyclonal C6219, 1:8000 dilution), anti β1 integrin (Santa Cruz Biotechnology, rabbit polyclonal sc-8978 1:200), anti-vinculin (Sigma-Aldrich, mouse monoclonal V4505, 1:2000 dilution), anti-α-tubulin (Sigma-Aldrich, mouse monoclonal T5168, 1:1000 dilution), anti-GAPDH (Cell Signaling Technology, rabbit monoclonal 2118, 1:1000 dilution). The membranes were then incubated with the appropriate AP- or HRP-conjugated secondary antibody (rabbit anti-mouse A4312 Sigma-Aldrich, 1:3000 dilution; donkey anti-rabbit NA9340V GE Healthcare, 1:3000 dilution) for 1 hour at room temperature. Immunocomplexes were detected using a Western blot chemiluminescent reagent (CDP-star, PerkinElmer or ECL western blotting detection reagents, GE Healthcare) following the manufacturer’s instructions. The densitometric evaluation of the bands was performed with AIDA software (Advanced Image Data Analyzer 2.11 Raytest GmbH, Germany).

### Immunofluorescence assays and actin cytoskeleton distribution pattern analyses

Cells were fixed in 4% paraformaldehyde (PFA) in PBS for 10 minutes at 4°C. Fixed cells were firstly permeabilized with Ethanol: Acetone 1:1 for 10 minutes at 4°C and then with 1% BSA, 0.1%-Triton X-100 in PBS for 1 hour at room temperature. Non-specific antibody binding was blocked for 30 minutes with 1% BSA, 0.1% Triton X-100, 5% donkey serum (Jackson ImmunoResearch Laboratories) in PBS.

Cells were incubated overnight in 1% BSA/0.1% Triton X-100 in PBS at 4°C with the following primary antibodies: anti-E-cadherin (Santa Cruz Biotechnology, rabbit polyclonal sc-7870 1:50), anti-β-catenin (Santa Cruz Biotechnology, mouse monoclonal sc-7963, 1:50 dilution), anti-connexin-43 (Sigma-Aldrich, rabbit polyclonal C6219, 1:50 dilution), and anti-p-Histone H3 (Santa Cruz Biotechnology, mouse monoclonal sc-374669, 1:50 dilution). The samples were then rinsed 3 times (30 minutes total) in 1% BSA/0.1% Triton X-100 in PBS and incubated with the opportune secondary antibody (FITC–conjugated donkey anti-rabbit 711-095-152 or donkey anti-mouse 715-095-150 IgG, Jackson ImmunoResearch Laboratories, 1:200 dilution) in PBS for 90 minutes at room temperature. Cells were washed in three changes of PBS (30 minutes total), mounted in buffered glycerol (0.1 M, pH 9.5) and photographed with a Zeiss fluorescent microscope. Negative controls were treated with secondary antibody only.

For F-actin visualization Rhodamine Phalloidin (Invitrogen Molecular Probes, Eugene, 1:400 dilution) was used. Briefly, 4% PFA fixed cells were permeabilized in cold Ethanol: Acetone 1:1 and incubated with Rhodamine Phalloidin for 25 min in the dark. Cells were then washed in PBS (three changes of 10 minutes), mounted in buffered glycerol (0.1 M, pH 9.5) and photographed with a Zeiss fluorescent microscope.

### E-cadherin and β-catenin RNA inteference (RNAi)

E-cadherin and β-catenin were knocked down, in control and EW treated TCam-2 cells, using the TriFECTa^TM^ Dicer-Substrate RNAi kit (Integrated DNA Technologies) following the manufacturer’s instructions. This kit contains three Dicer substrate 27-mer duplexes for each target gene. TCam-2 cells were treated overnight, 24 hour after plating, with these DsiRNA cocktails (0.18 nM each DsiRNA) against E-cadherin or against β-catenin or with same concentration of negative control duplex, together with lipofectamine (Lipofectamine 2000 reagent invitrogen) diluted 1:400 in Opti-MEM reduced serum medium. Cell treatment with lipofectamine alone was also used as additional internal control. Duplexes internalization efficiency was evaluated using TYE^TM^ 563 DC transfection control that is a fluorescent-labeled control duplex: it was estimated as 82.78%+ 2.56(SEM) of all cultured TCam-2 cells without significant differences between control and EW treated samples. Forty-eight hours after RNAi treatment cells were starved overnight and then harvested, counted and dead cells were calculated using trypan blue exclusion staining technique. In all the samples the percentage of dead cells is always less than 9% and no statistical differences were observed between samples. Harvested cells were partly used for western blot analyses of E-cadherin and β-catenin to evaluate silencing efficacy, and partly added to Boyden’s upper chamber (7.5 X 10^4^ cells/chamber) for Boyden chamber assay (see below). E-cadherin and β-catenin western blot analyses performed after RNAi were densitometrically evaluated using AIDA software (Advanced Image Data Analyzer 2.11 Raytest GmbH, Germany). Lipofectamine and negative control duplex did not affect E-cadherin or β-catenin protein content (not shown).

### Boyden chamber assays

The cells were assayed for their ability to migrate through a polycarbonate filter (pore size, 8 µm; Whatman International) using Boyden chambers (NeuroProbe) as previously described [25]. Cells were cultured for 72 hours with or without 10% EW in the presence of 10% FBS and then maintained for 16 hours under serum-free conditions. Cells were then trypsinized, re-suspended in serum-free RPMI 1640 containing 0.1% BSA and added to the upper chamber (7.5 X 10^4^ cells/chamber). As chemo-attractant, FBS (1% to 5%) was added in the lower chamber. Migration was allowed to progress for 5 hours at 37°C in a humidified atmosphere with 5% carbon dioxide. Filters were then fixed and stained. Cells from the upper side of the filter were carefully removed using a cotton swab. Migrated cells present in the lower side of the filters were visualized and counted by bright-field microscopy (Zeiss Axioscope) using a 40x objective and the average number of cells per field was calculated. Data are expressed as a migration index and calculated as the fold increase over the control. The results are reported as the means ± standard error (SEM).

### Wound healing assay

Wound healing assays were performed using silicone Ibidi Culture-Inserts (Ibidi 80209). The cells were cultured for 72 hours with or without 10% EW in the presence of 10% FBS and maintained for 8 hours under serum-free conditions. Cells were then trypsinized, counted, plated into each well of the culture-inserts (7x10^4^ cells per well; that is 3.18x10^5^/cm^2^) and incubated at 37°C in a humidified atmosphere with 5% carbon dioxide. After 15 hours of cell attachment, the culture-inserts were gently removed. The gap was periodically monitored and the images were recorded. Wound healing assay was allowed to proceed for 72 hours. Images were analysed to measure the open wound area using the TScratch Software developed by the Koumoutsakos group (CSE Lab), at ETH Zürich [26].

To asses proliferation rate of cells cultured in wound healing assay condition, we performed immunofluorescences of the M-phase marker p-Histone H3, as described previously in the immunofluorescence paragraph, at 24 and 48 hours of culture after Ibidi Culture-Inserts removal.

### Image morphometric analysis

To perform quantitative morphometric analysis of TCam-2 cell shape single cells from F-actin detection images were contoured with a fine black marker by different researchers, scanned, and catalogued according to the different culture conditions (Table 1).

**Table 1:** Number of cells analyzed for each group and time point.

**Time (h)**	**Control (Number of cells analysed)**	**EW (Number of cells analysed)**
24	40	40
48	40	40
72	214	290

The random extraction of 40 cells in the cases 72 (A and B) hours shows a significance compared to the total number cells, so we can select only 40 cells in the cases 24 (A and B) and 48 (A and B) hours.

All the images were processed by Photoshop CS4. Black contoured cell edges were refined. Cells were then black filled and threshold was adjusted to remove other cells and background from image.

A single cell sheet was generated at each time point. To obtain single cell shape parameters: Circularity, Solidity and the Fractal Dimension (evaluated using the box counting method, FracLac plugin), ImageJ V1.47h software was used. To calibrate the software, all the pictures were resized according to original scale of image acquisition.

Subesequently, the software analysed single cells, providing shape parameters. In addition to area A and perimeter P, the following parameters were calculated:

Circularity = 4πA/(2P)^2^

Solidity = A/CA

Where CA is the convex area.

For each parameter a macro was created, and results were saved in Microsoft Excel format, to obtain the mean and relative standard error for each parameter.

Finally, single graphs of Fractal Dimension, Circularity, and Solidity were obtained for each set of images.

Computations were all performed blindly.

### Transmission Electron Microscopy

TCam-2 cells, cultured in the presence or absence of 10% EW, were fixed in 2.5% Glutaraldehyde in 0.1 M Cacodylate Buffer (pH 7.4), postfixed in 1% OsO_4_, treated with 1% Tannic Acid, de-hydrated in Ethanol and embedded in epoxy resin. Ultrathin sections were contrasted in aqueous lead-hydroxide followed by Tannic Acid treatment and photographed by a Hitachi 7000 Transmission Electron Microscope (TEM).

### Statistical analysis

All quantitative data are presented as the mean value ± standard error (SEM). Student t-test and ANOVA followed by Duncan test for multi-group comparison were carried out, where appropriate, to evaluate the significance of differences. The significance level was fixed at a P value < 0.05.

## Results

### EW does not affect TCam-2 cell proliferation and survival

We tested the ability of EW to modify seminoma cell proliferation and survival by culturing TCam-2 cells in the presence or absence of 10% EW for 24, 48 and 72 hours. We found that the total number of cells, as well as the number of dead cells, does not vary in EW-cultured cells compared with the respective control conditions (Table 2).

**Table 2:** Proliferation and survival analyses of TCam-2 cells cultured with or without EW.

**Viable Cells (X10^5^)/cm^2^ ± SE**	**Time 0**	**24h**	**48h**	**72h**
**Control**	0,36	1,09 ± 0,01	1,53 ± 0,02	3,34 ± 0,19
**EW**	0,36	1,09 ± 0,01	1,54 ± 0,002	3,82 ± 0,42
**Percentage of Dead Cells/cm^2^ ± SE**				
**Control**		0,75 ± 0,01	0,48 ± 0,09	0,79 ± 0,15
**EW**		0,62 ± 0,08	0,58 ± 0,04	0,80 ± 0,03

The number of viable cells and the percentage of trypan blue stained dead cells at each time point is shown. Data are shown as the mean±SEM of triplicate samples. Statistical significance was evaluated by Student’s T test. Values between control and EW cultured cells are not significant.

In addition, FACS analysis was performed to determine the percentage of TCam-2 cells in all the cell cycle phases. Again, we found no significant differences between control and treated cells at all time points (Table 3).

**Table 3:** Cell cycle analysis of TCam-2 cells cultured with or without EW.

**G1 Phase (% Cells)**	**24h**	**48h**	**72h**
**Control**	59,8 ± 0,1	57,9 ± 1,5	61,3 ± 0,1
**EW**	59,4 ± 1,4	58,7 ± 0,8	60,0 ± 1,0
**S Phase (% Cells)**			
**Control**	28,1 ± 0,1	28,5 ± 1,7	27,1 ± 0,1
**EW**	28,3 ± 0,3	27,2 ± 0,7	28,4 ± 1,2
**G2 Phase (% Cells)**			
**Control**	12,2 ± 0,1	13,6 ± 0,2	11,7 ± 0,19
**EW**	12,3 ± 0,1	14,2 ± 0,04	11,6 ± 0,42

Flow cytometry analysis of cell cycle phases distribution of TCam-2 cells cultured in the absence or presence of 10% EW for 24, 48, and 72 hours. Data are shown as the mean±SEM of triplicate samples. Statistical significance was evaluated by Student’s T test. Values between control and EW cultured cells are not significant.

### EW increases TCam-2 seminoma cell-substrate adhesion and spreading rate

**Figure 1:**
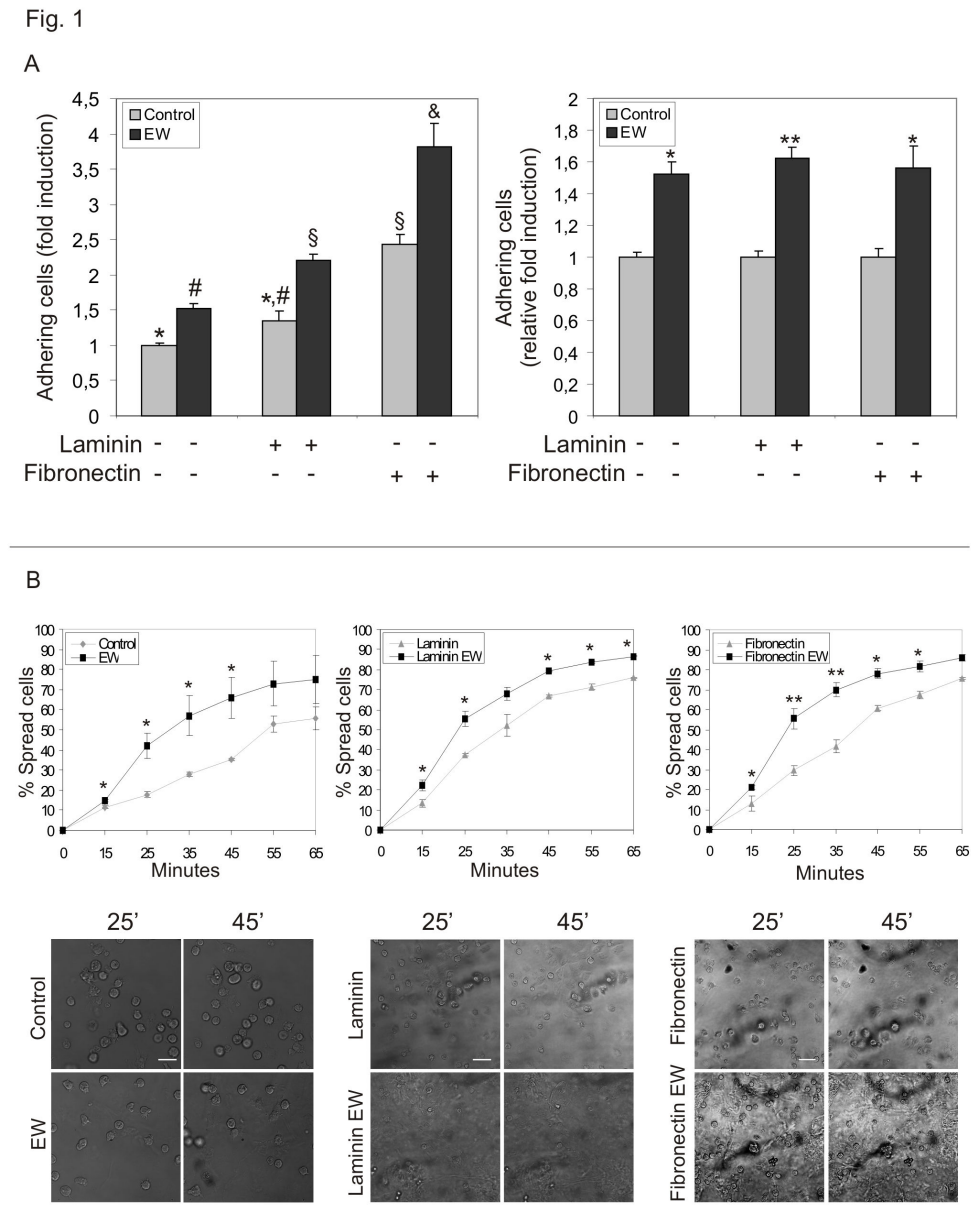
EW increases TCam-2 adhesive behaviour. A) Adhesion assay of TCam-2 cells cultured in the absence or presence of 10% EW for 40 minutes on plastic dishes and on laminin or fibronectin coated plastic dishes. In the left graph the values are expressed as fold induction compared to control condition on plastic (arbitrary referred as 1). Statistical significance was evaluated by ANOVA test. Same symbols (n.s.). Different symbols (P<0.05). In the right graph the values are expressed as relative fold induction considering as 1 the number of adhering cells of each relative control condition. Statistical significance was evaluated by Student’s T-test. * *vs* its control P<0.05. ** *vs* its control P<0.01. B) Video time lapse analysis of TCam-2 cell spreading capability. TCam-2 cells, cultured on plastic or on laminin or fibronectin coated plastic dishes were allowed to spread in the presence or absence of 10% EW for 50 minutes (Upper panel). graphical presentation of the percentage of TCam-2 spread cells in control and EW-cultured samples at different time points and on different substrates. Statistical significance was evaluated by ANOVA test. * *vs* its relative control P<0.05. ** *vs* its relative control P<0.01 (Lower panel). representative images of TCam-2 cell spreading at different time points and on different substrates. One representative of three experiments performed is shown. Bar, 50 µm.

The ability of EW to modulate the rate of cell-substrate adhesion was tested culturing TCam-2 cells for 40 minutes on plastic dishes, pre-coated or not with laminin or fibronectin, in presence or in absence of 10% EW. We chose laminin as basal lamina substrate and fibronectin as stromal extracellular matrix adhesive substrate. Both molecules are proper of seminoma niche [27]. At the end of the culture time, we counted adherent cells and we found that, even if the percentage of adherent cells cultured in control condition on fibronectin is significantly higher compared to the plastic and laminin respective control samples (Figure 1A left panel), the percentage of adherent TCam-2 cells is always significantly higher in EW treated samples, compared to their relative control, regardless the adhesive substrate considered (Figure 1A right panel).

On the basis of these observations, we tested whether EW could also induce a faster TCam-2 cell spreading. Hence, TCam-2 cells were cultured on plastic dishes, pre-coated or not with laminin or fibronectin, with or without 10% EW for 50 minutes at 37°C and, during this culture period, a computerized time-lapse video analysis was performed. Differences between treated and control samples were observed already at 10 minutes of incubation and TCam-2 cell spreading resulted faster in the presence of EW than in control cells in all the substrate culture condition considered (Figure 1B upper panel). Images collected from a representative time lapse analysis at 25 and 45 minutes were reported in Figure 1B (lower panel). As shown, round cells decreased in number faster in EW treated samples than in control conditions.

### EW does not affect TCam-2 β1 integrin and vinculin protein content

**Figure 2:**
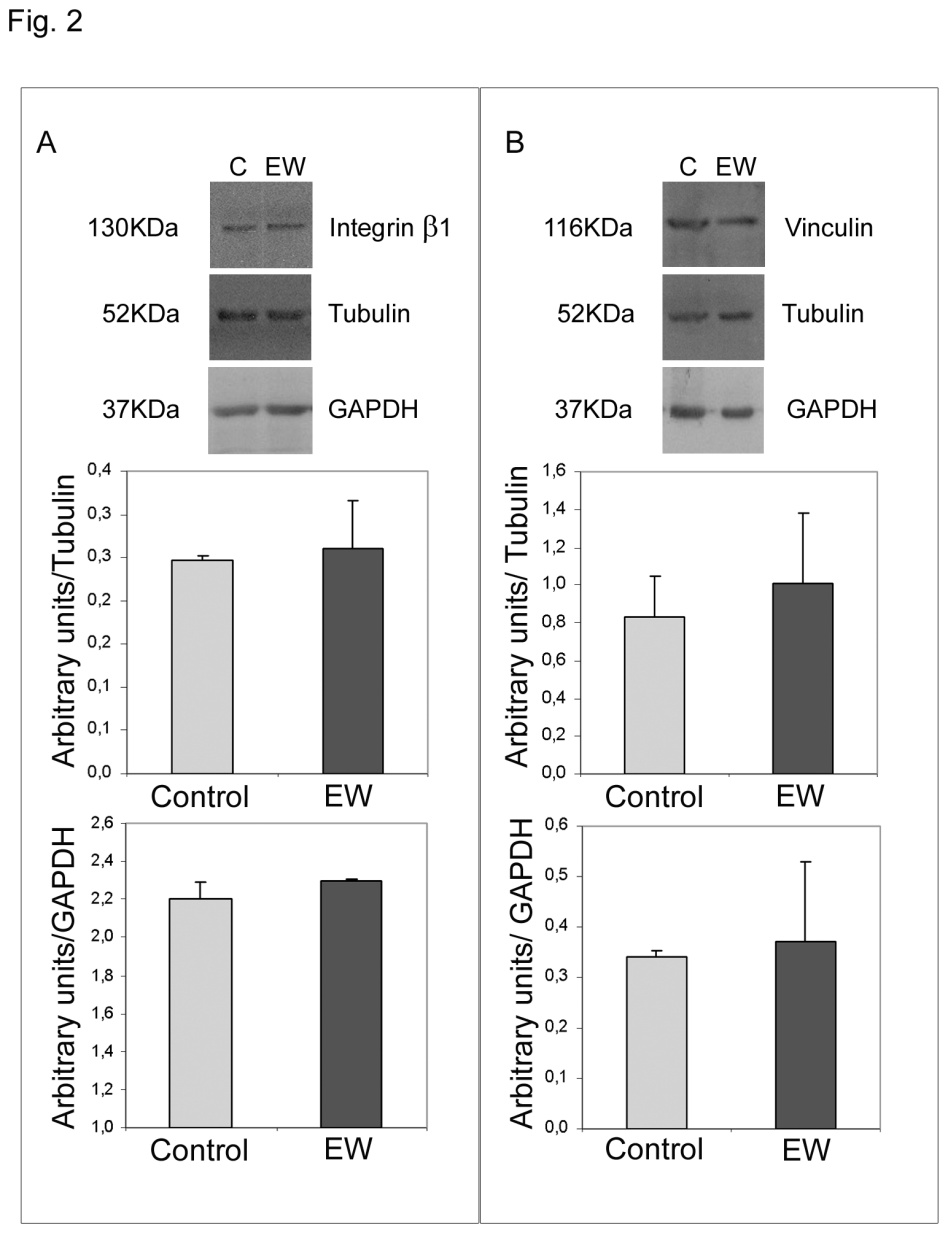
EW does not influence β1 integrin subunit and viculin protein content. A) Western blot analysis of β1 integrin subunit in TCam-2 cells cultured with or without 10% EW for 72 hours. As expected a 130kDa band was detected by immunoblot analysis. The densitometric analyses of the bands, normalized *versus* tubulin and *versus* GAPDH, observed in three independent experiments, are reported. Statistical analysis, evaluated by Student’s T test, was not significant. B) Western blot analysis of vinculin in TCam-2 cells cultured with or without 10% EW for 72 hours. As expected a 116kDa band was detected by immunoblot analysis. The densitometric analyses of the bands normalized *versus* tubulin and *versus* GAPDH, observed in three independent experiments, are reported. Statistical analysis, evaluated by Student’s T test, was not significant.

To assess whether EW administration could induce specific cell-substrate adhesion machinery we aimed to investigate the protein amount of β1 common chain of VLA integrins and the cytoplasmic membrane cytoskeleton adaptor protein vinculin. Both proteins are known to be expressed in normal testis [28] and in particular β1 integrin chain (that forms both a laminin and a fibronectin receptor) has been established as one of the integrin subunit mainly expressed in TGCTs [27]. Western blot analyses allowed us to observe that neither β1 integrin nor vinculin appeared to be regulated by EW treatment (Figure 2). These data are in line with the previous reported adhesion assay observations where the relative induction of cell adhesion after EW administration does not change in percentage when compared with the respective control samples regardless the adhesive substrate used (Figure 1A right panel).

### EW is able to modify TCam-2 cell morphology and F-actin distribution pattern: a molecular and morphometric analysis

**Figure 3:**
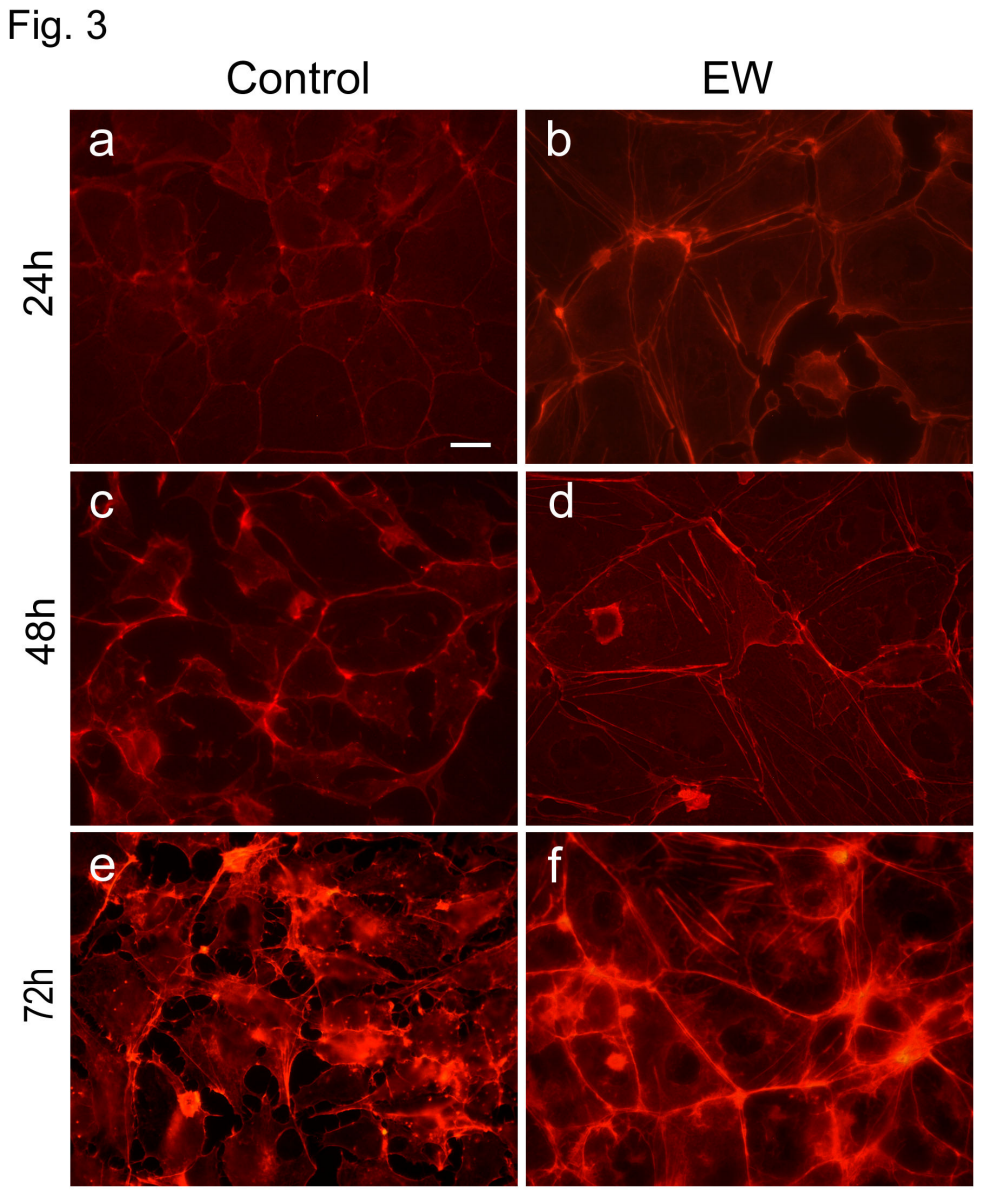
EW changes F-actin distribution pattern. Rhodamine-phalloidin staining of TCam-2 showing F-actin distribution pattern after 24, 48, and 72 hours of culture in the presence or absence of 10% EW. In a, c, and e control cells are shown whereas in b, d, and f EW cultured cells are shown. Bar, 20 µm.

**Figure 4:**
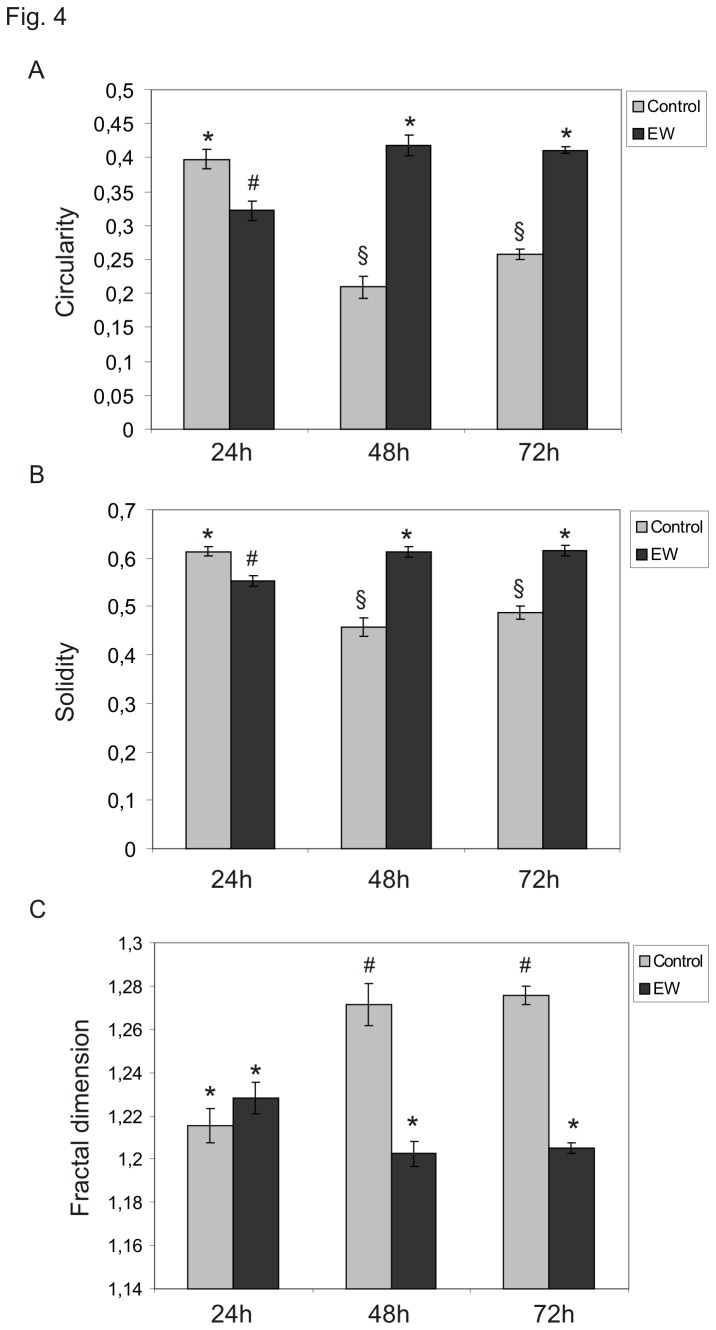
EW modifies TCam-2 cell shape. A) Graphical representation of Circularity parameter variation in TCam-2 cells cultured for 24, 48, 72 hours. Statistical significance was evaluated by ANOVA test. * *vs* # P< 0.001; * vs § P< 0.001; # vs § P< 0.05. Same symbols indicate no statistical significance. B) Graphical representation of Solidity parameter variation in TCam-2 cells cultured for 24, 48, 72 hours. Statistical significance was evaluated by ANOVA test. * *vs* # P< 0.001; * *vs* § P< 0.001; # *vs* § P< 0.05. Same symbols indicate no statistical significance. C) Graphical representation of Fractal Dimension variation in TCam-2 cells cultured for 24, 48, 72 hours. Statistical significance was evaluated by ANOVA test. * *vs* # P< 0.001. Same symbols indicate no statistical significance.

TCam-2 cells growing in EW-additioned culture medium undergo a dramatic modification in cell shape. The majority of control cells showed an irregular morphology characterized by multiple membrane protrusions, while EW treated cells appear mainly polygonal, and apparently more closely attached to each other. Thus, in order to better characterize these differences in cell shape, we evaluated TCam-2 cells F-actin cytoskeleton modification, in presence or absence of EW for 24, 48 and 72 hours. As expected, a significant modification in the F-actin cytoskeleton distribution pattern was observed in EW-cultured cells compared with the control (Figure 3). After 24 hours of culture, EW-cultured samples showed actin filaments well ordered in cortical bundles corresponding to cell-cell contacts and this distribution pattern became more evident as the culture time elapsed. Interestingly, after 48 hours of culture even actin stress fibres began to be detectable in the EW treated samples. These findings indicated that EW administration caused evident cytoskeleton remodelling. Morphometric evaluation of Circularity and Solidity of cultured cells with and without EW showed the increase of both these parameters in EW treated cells. The increase became evident at 48 and 72 hours of culture, according to the contemporary observed cytoskeleton modifications (Figure 4 A,B).

The irregular geometry of cultured cells was then measured by calculating the Fractal Dimension, a parameter inversely related to Circularity and Solidity. As expected decrease was observed in Fractal Dimension after EW administration at 48 and 72h, thus confirming solidity and circularity data (Figure 4 C).

The data presented indicate how, in recovering a more rounded profile (low Fractal Dimension), EW-cultured cells have a more regular shape (Circularity), and a reduced deformability (Solidity). A reduction in FD suggests that the neoplastic cells partly lose their invasiveness, considering that high FD values are associated to a "diffusive" shape (the form the cell acquires when it displays an invasive pattern), that is the stage that precedes the metastatic spreading [29].

### EW up-regulates E-cadherin, β-catenin and connexin-43 and modifies TCam-2 junctional capability

On the basis of the morphological modifications observed under EW treatment, we assessed whether EW can modify TCam-2 cell-cell adhesive properties. Thus we aimed to investigate the protein amount and the distribution pattern of E-cadherin, β-catenin and connexin-43.

**Figure 5:**
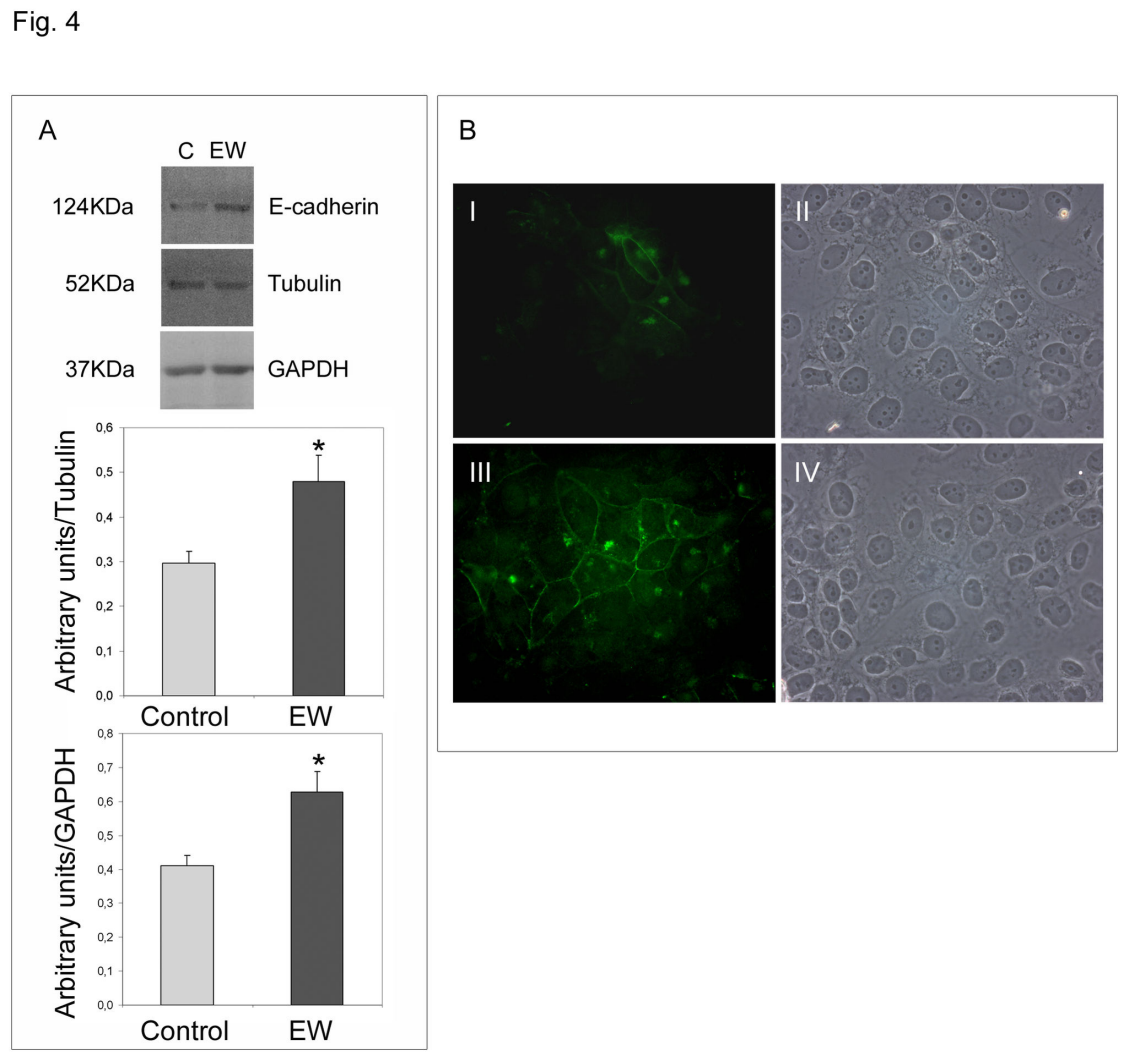
EW up-regulates E-cadherin expression in TCam-2 cells. A) Western blot analysis of E-cadherin in TCam-2 cells cultured with or without 10% EW for 72 hours. As expected a 124kDa band was detected by immunoblot analysis. The densitometric analyses of the bands, normalized *versus* tubulin and *versus* GAPDH, observed in three independent experiments, are reported. Statistical significance was evaluated by Student’s T test. * vs. control, P< 0.05. B) E-cadherin immunolocalization in TCam-2 cells cultured in absence (I) or in the presence of 10% EW (III) for 72 hours. In II and IV the respective bright fields are reported. Bar, 20 µm.

**Figure 6:**
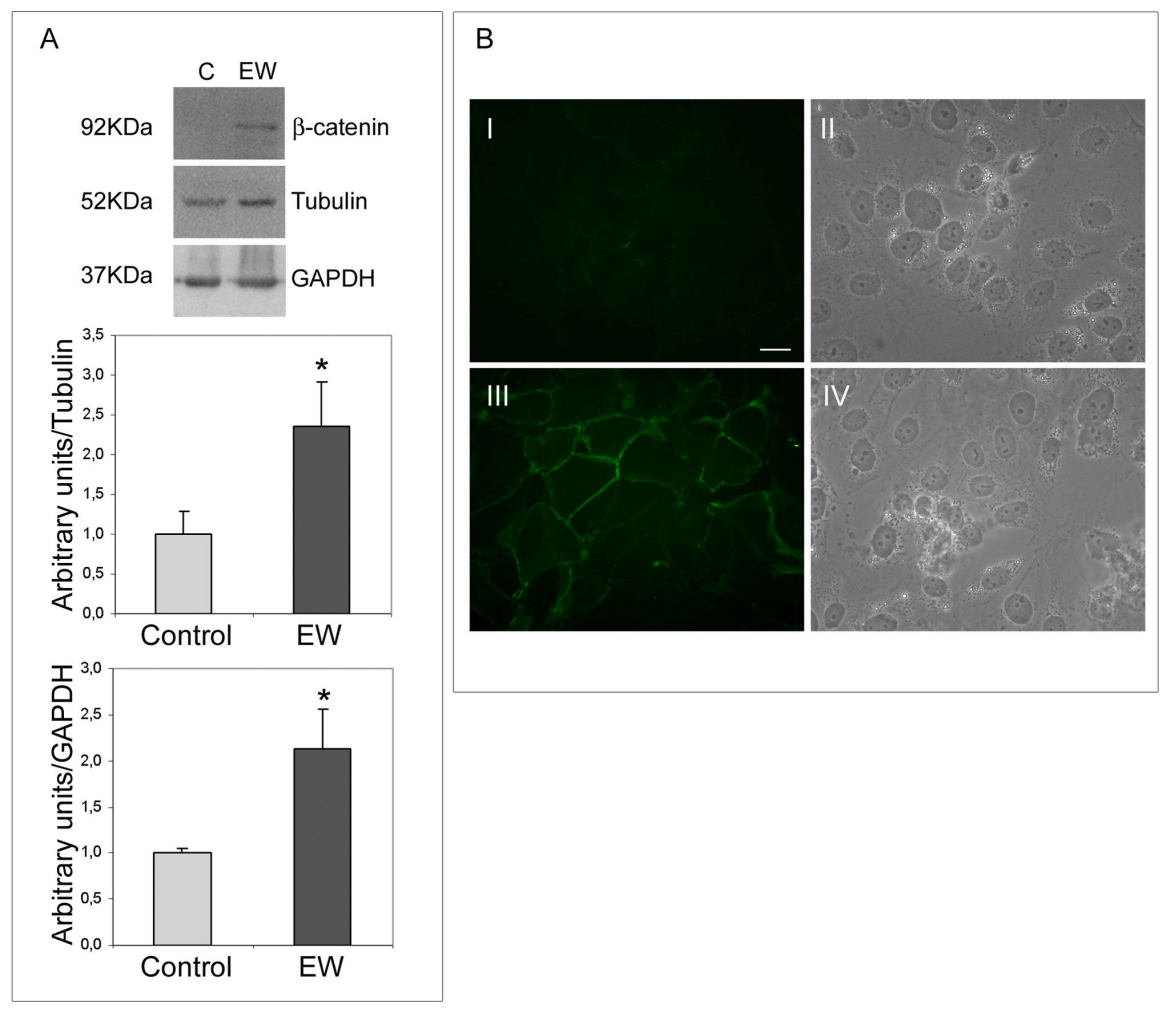
EW up-regulates β-catenin expression in TCam-2 cells. A) Western blot analysis of β-catenin in TCam-2 cells cultured with or without 10% EW for 72 hours. A 92 kDa band corresponding to the predicted β-catenin molecular weight was detected by immunoblot analysis. The densitometric analyses of the bands, normalized *versus* tubulin and *versus* GAPDH, observed in three different experiments, are reported. Statistical significance was evaluated by Student’s T test. * vs. control, P< 0.05. B) Immunodetection of β-catenin in TCam-2 cells cultured in absence (I) or in the presence of 10% EW (III) for 72 hours. In II and IV the respective bright fields are reported. Bar, 20 µm.

**Figure 7:**
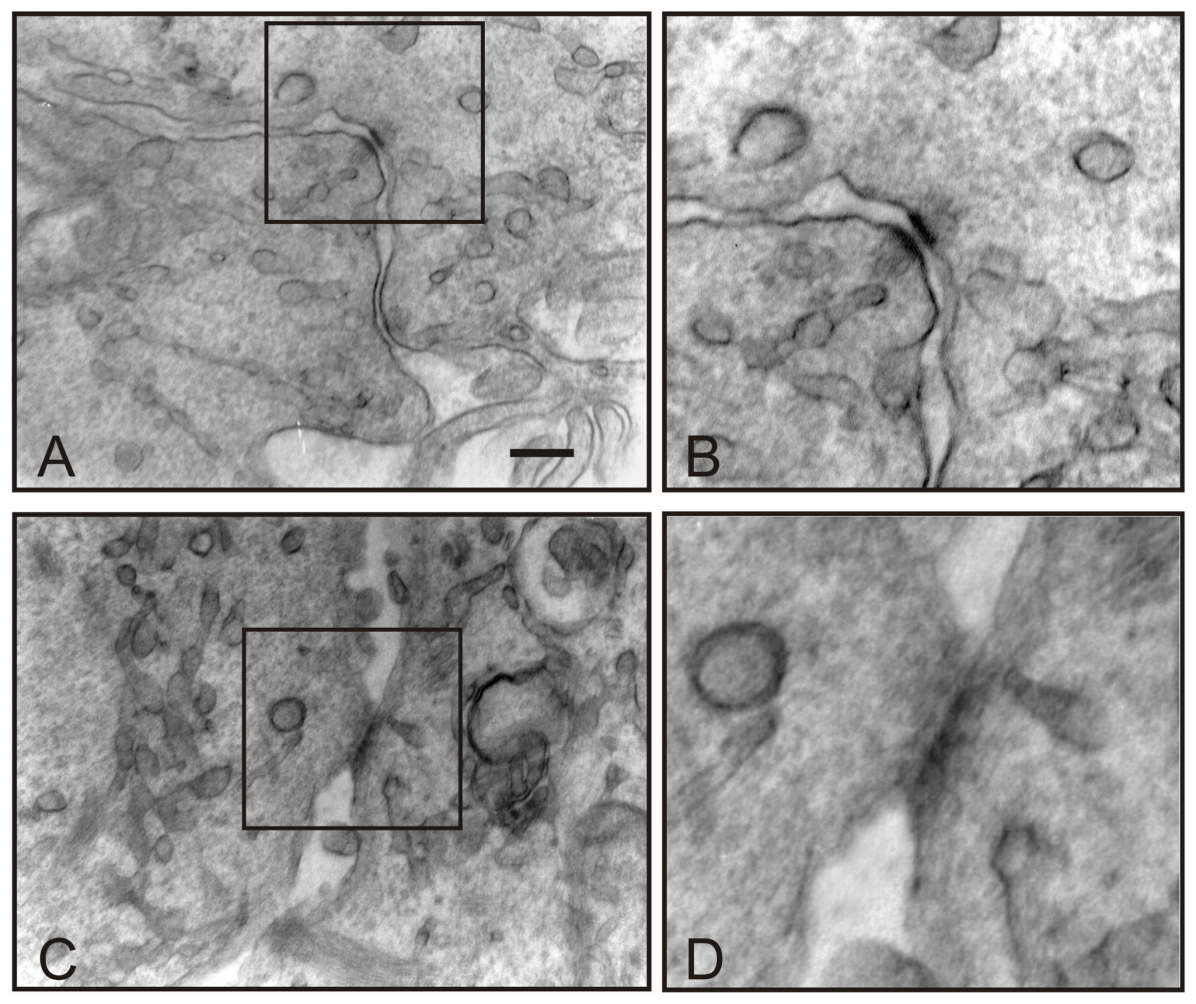
EW induces junctional contact formation in TCam-2 cells. Transmission electron microscopy pictures showing EW induced TCam-2 cell-cell junctional contacts. In A one typical cell-cell adhesion junction is showed and in B an higher magnification of the same junction is reported. In C cellular contact compatible with gap-junction structure is shown and in D a higher magnification of the same junction is reported. Bar, A and C 226 nm B and D: 150 nm.

The adherens junction-related proteins E-cadherin and β-catenin appear to be up-regulated by EW (Figures 5A, 6A). The immunofluorescence analysis revealed that both E-cadherin and β-catenin were barely detectable in control samples, whereas in EW administered samples scattered groups of cells exhibited a strong cortical signal for both proteins (Figures 5B, 6B). Since E-cadherin is involved in junctional adherent complexes, and it is linked to the actin cytoskeleton through its interaction with β-catenin, we hypothesize that the E-cadherin and β-catenin expression modulation may be involved in EW-induced actin cytoskeleton remodelling. Indeed, TEM analyses clearly revealed the de novo formation of adherens junctional complexes in the EW treated samples only (Figure 7A, B).

**Figure 8:**
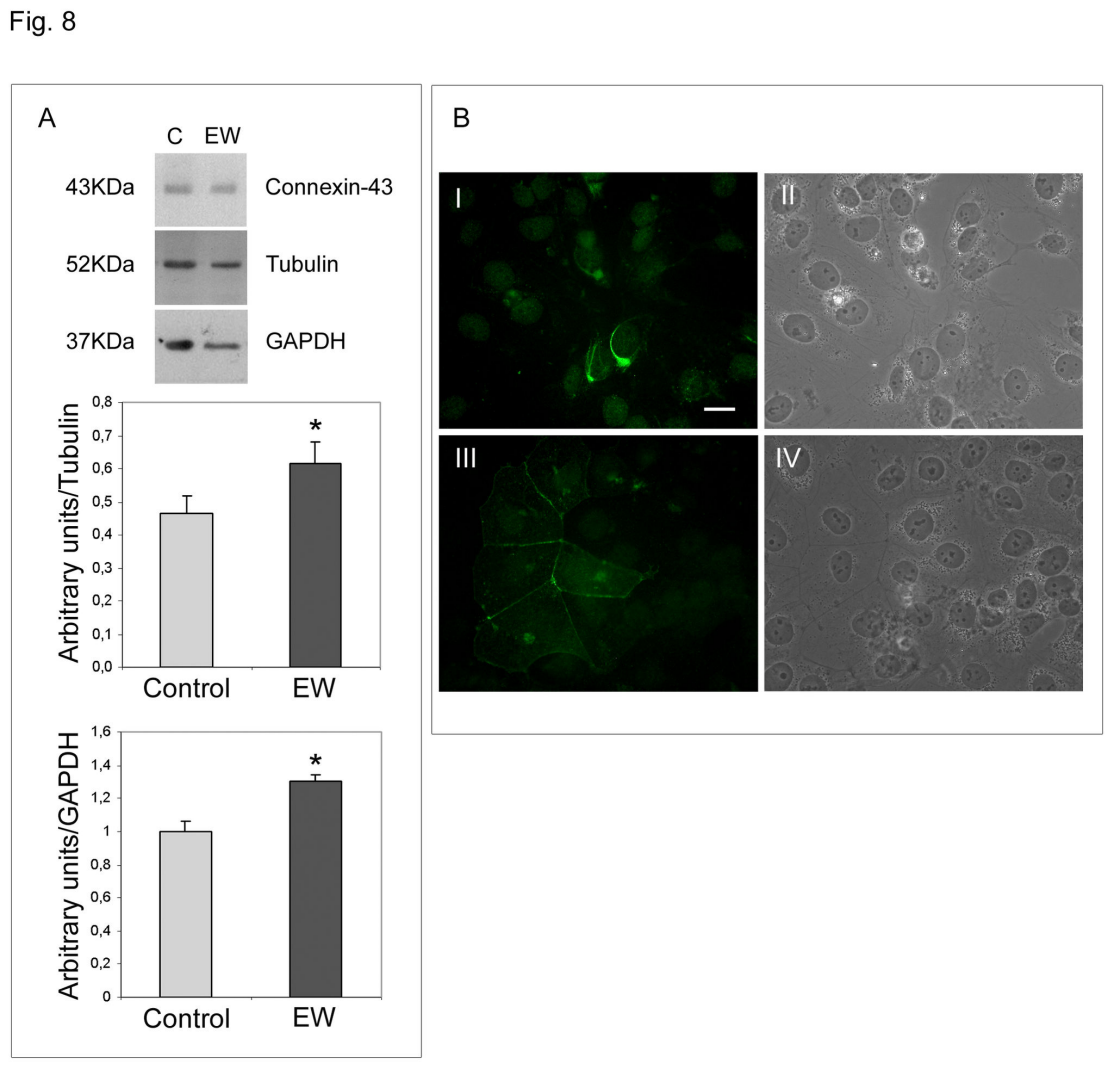
EW up-regulates connexin-43 expression and influences its distribution pattern in TCam-2 cells. A) Western blot analysis of connexin-43 in TCam-2 cells cultured for 72 hours in control medium or in 10% EW supplemented medium. As expected anti-connexin-43 detected one band of 43 kDa. The densitometric analyses of the bands, normalized *versus* tubulin and *versus* GAPDH, from three different experiments, are reported. Statistical significance was evaluated by Student’s T test. * vs. control, P< 0.05. B) Immunofluorescence staining of connexin-43 in TCam-2 cells cultured in absence (I) or in the presence of 10% EW (III) for 72 hours. In II and IV the respective bright fields are reported. Bar, 20 µm.

The gap-junction related protein connexin-43 also appears to be up-regulated by EW (Figure 8A). Immunofluorescence analysis revealed that connexin-43 in control cells appeared to be distributed in the perinuclear compartment, whereas EW-cultured cells showed a typical connexin-43 membranous staining (Figure 8B), sustaining the establishment of well-organized gap junctional complexes, that were recovered, as expected, only from EW treated samples. Transmission Electron Microscopy analysis revealed the presence of structures compatible with gap junctional complexes only in the EW treated samples (Figure 7 C, D).

### EW inhibits TCam-2 cell migration

**Figure 9:**
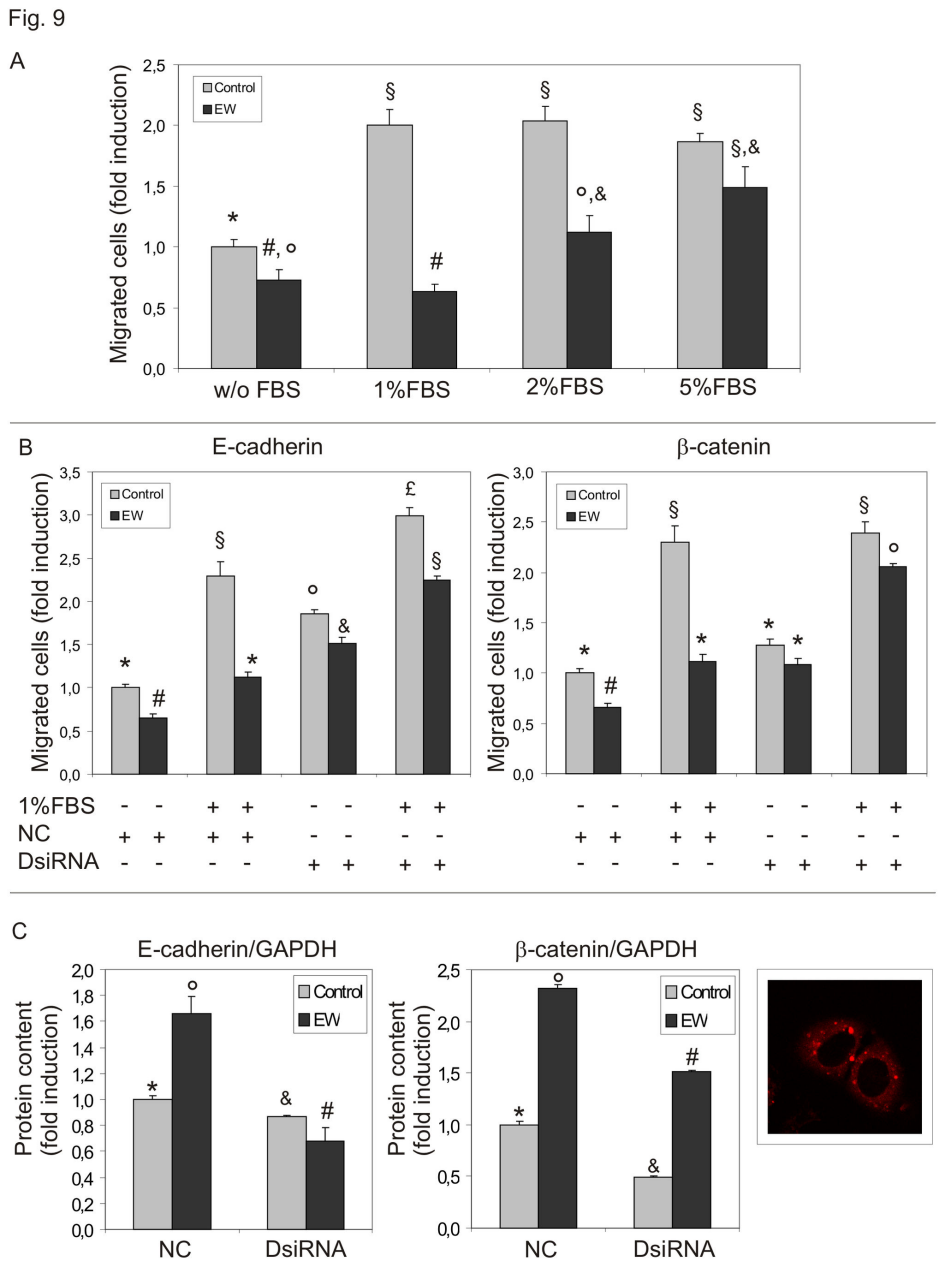
EW alters TCam-2 cell chemo-sensitive dependent migration. A) Boyden chamber assay evaluation of FBS induced cell migration of TCam-2 cells. Increasing concentrations of FBS were used as chemoattractant. EW was used to treat cells before the migration assay when indicated. Data are expressed as the mean ± SEM (n=3, measured in triplicate). Statistical significance was evaluated by ANOVA test Same symbols indicate no significance. Different symbols indicate P<0.05. B) Boyden chamber assay evaluation of TCam-2 cell migration after E-cadherin or β-catenin silencing. EW was used to pre-treat cells when indicated. Data are expressed as the mean ± SEM (n=3, measured in triplicate). Statistical significance was evaluated by ANOVA test. Same symbols indicate no significance. Different symbols indicate P<0.05. C) Left panel: densitometric analyses of western blots after E-cadherin andβ-catenin RNAi. The values are expressed as fold induction compared to negative control duplex (NC) of control (EW-untreated) TCam-2 cells (arbitrary referred as 1). Statistical significance was evaluated by ANOVA test. Same symbols indicate no significance. Different symbols indicate P<0.05. Right panel: representative image of lipofectamine treated TCam-2 cells cultured with the red fluorescent-labeled control duplex: cytoplasmic internalization of fluorescent double strand RNA (red dots) is clearly observable.

To test the hypothesis that EW could affect TCam-2 chemo-polarized migration, we performed Boyden chamber migration assays. Increasing concentrations of FBS (1–5%) were used as chemoattractant. EW significantly impaired TCam-2 cell migration and even more interestingly, EW was also able to almost completely abolish 1-2% FBS induced cell migration. However, no significant effects were observed with 5% FBS concentrations (Figure 9A).

We performed Boyden Chamber assays also using E-cadherin or β-catenin silenced TCam-2 cells. Interestingly E-cadherin knock-down was able to increase significantly the migratory ability of TCam-2 seminoma cells even in control condition and is able to completely rescue the migratory capability of EW treated sample. Also β-catenin knock-down is able to rescue migratory behaviour of EW treated TCam-2 cells even if it does not reach the same level of negative control duplex EW untreated samples (Figure 9B). This result can be easily explained considering that β-catenin knock-down in EW treated samples was less efficient than the E-cadherin knock-down in the same samples (Figure 9C). These data indicate that both E-cadherin and β-catenin mediated cell to cell adhesion is crucial for seminoma cell stability and likely the up-regulation of these proteins may prevent seminoma metastatic behaviour.

**Figure 10:**
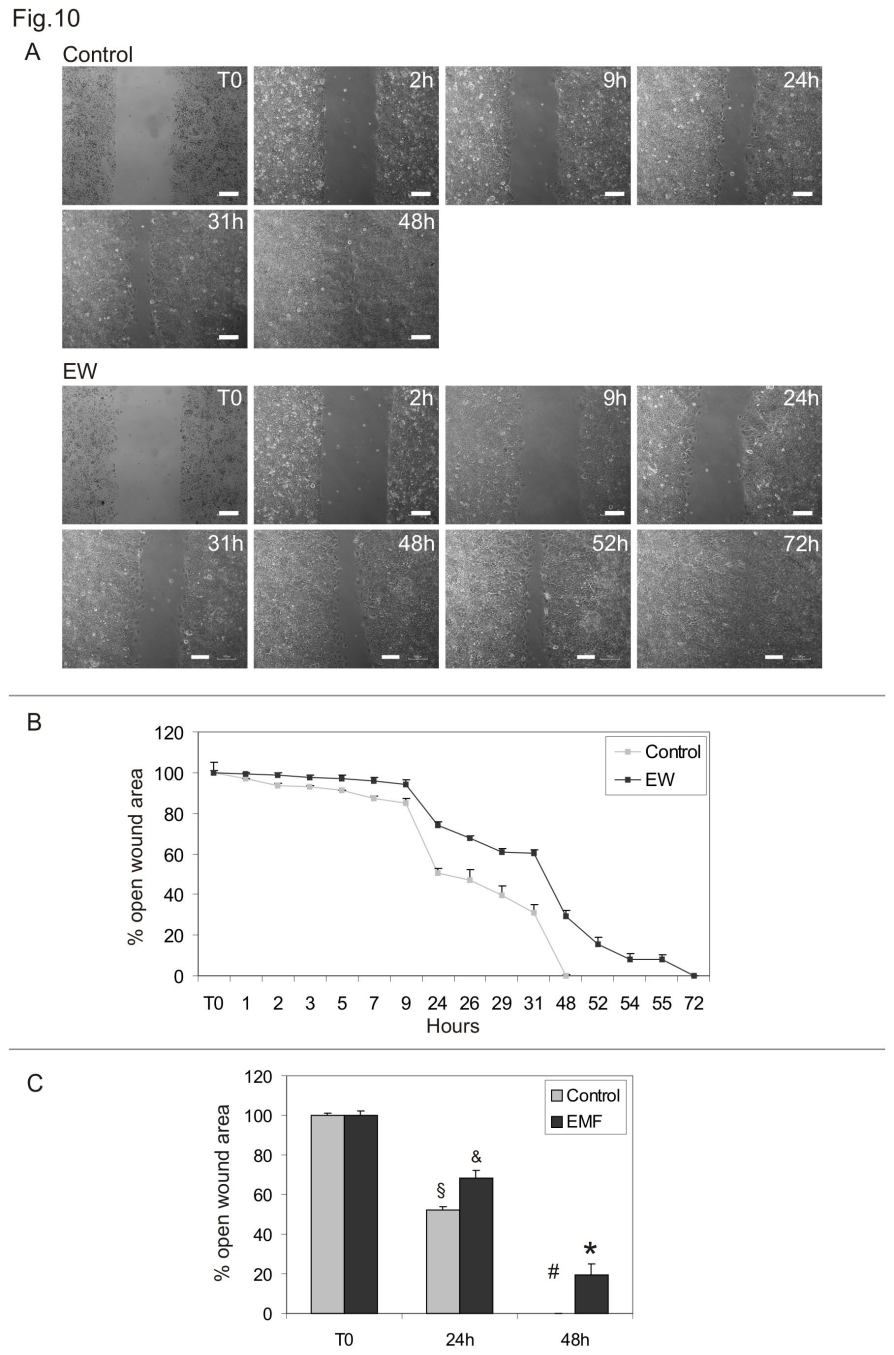
EW modifies TCam-2 cells collective migration. Cell wound healing assay. A) Representative phase-contrast microscopy of TCam-2 control cells and EW treated cells of one representative experiment at different time points after the beginning of wound healing assay. Bars, 100 µm. B) Graphical representation of the percentage of open wound area in control and EW treated cells from time 0 to 72 hours of culture. The values were normalized against the wound’s width at time 0. Values relative to one representative experiment performed in duplicate are shown. C) Graphical representation of the percentage of open wound area in control and EW treated cells at time 0, 24 and 48 hours of culture. The values were normalized against the wound’s width at time 0. The mean values of three independent experiments are shown. Statistical significance was evaluated by ANOVA test. Same symbols (n.s.). Different symbols (P<0.05).

**Figure 11:**
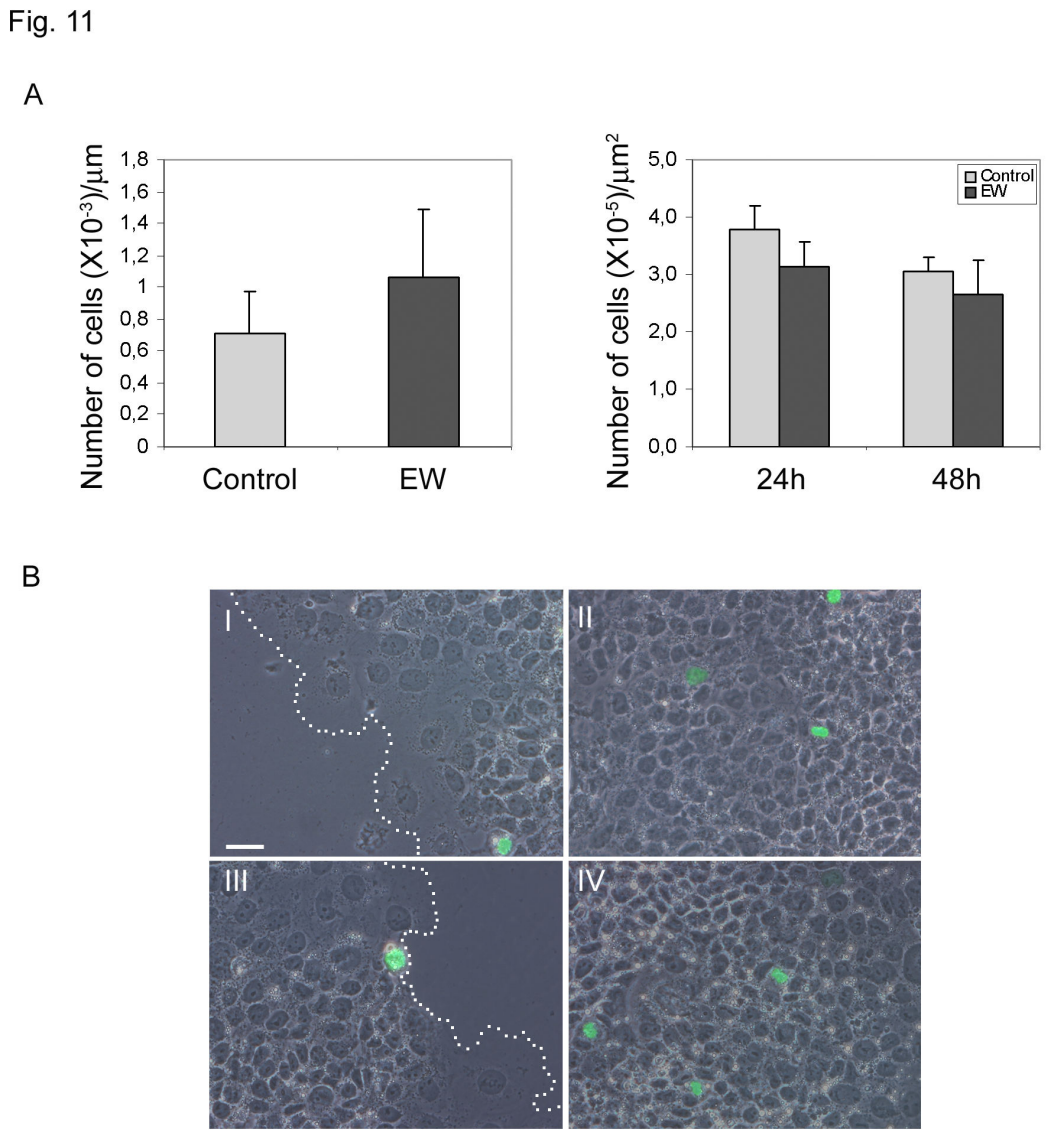
EW does not modify the number of pHistone H3 positive cells in the wound healing assays. A) Graphs indicating the number of pHistone H3 positive cells/µm at the wound’s edge (24 hours of culture; left graph) and the number of pHistone H3 positive cells/µm^2^ on area where cells are confluent (24 and 48 hours of culture; right graph). The number of pHistone H3 positive cells at 48 hours of culture at the wound’s edge were not reported since at this culture time control samples completely close the wound. Statistical analysis, evaluated by Student’s T test, was not significant. B) Images obtained merging phase contrast microscopy with pHistone H3 FITC immuno-detection. I and II represents images of control samples photographed at wound’s edge (I) and on area where cells are confluent (II). Dashed lines in I and II represent wound’s border. III and IV represents images of EW treated cells photographed at wound’s edge (III) and on area where cells are confluent (IV). Bar 50 µm.

Wound healing assays were conducted to evaluate the collective cell motility of TCam-2 cells cultured in the absence or presence of 10% EW. A wound was developed on a cell monolayer using silicone Ibidi Culture-Inserts and wound closure was assessed at various time points. EW hindered TCam-2 cells collective migration when compared with control cells (Figure 10). More precisely, control cells were able to close the wound after 48h of culture, while 57 to 72h were necessary for EW cultured cells. It is noteworthy that the number of mitotic cells, counted at 24 and 48 hours of culture at the edge of the wound as well as in the part of the dish where the cells have grown as monolayer, does not change significantly after EW administration (Figure 11).

## Discussion

The present investigation was inspired by the ‘embryonic rest’ theory of cancer, supported by several scientific studies that claim cancer stem cells derive from dormant embryonic remnants [1-4]. This theory gives rise to the intriguing possibility of creating differentiation therapies capable of reprogramming cancer stem cells simulating the appropriate embryonic microenvironment. Previous studies, from the 1970s to the present day, have documented the ability of embryonic microenvironments to reprogram aggressive cancer cells towards a less aggressive phenotype [10-17]. We studied TCam-2 seminoma cells considering that this particular cancer develops from Carcinoma in situ, a pre-invasive lesion with origin during the embryonic development because of the delayed or blocked differentiation of PGCs or gonocytes, due to an alteration of the testicular embryonic niche [6,7]. To provide embryonic cues we used a medium added with egg albumen (EW) whose microenvironment is able to regulate chicken embryo protein biosyntesis [30]. Firstly we found that EW modifies cell spreading rate and cell-substrate adhesion without affecting proliferation and survival indexes of TCam-2 cells in 40-50 minutes cultures. Modulation of cell adhesion to substrates is a key-step during the early phases of metastatic progression, when individual or small groups of cancer cells may acquire the ability to detach from a primary tumour. In particular integrin-extracellular matrix interplay has been reported as potentially important in Testicular Germ Cell neoplasia biological behaviour [27]. For this reason we assessed whether the EW-induced adhesion/spreading modulation could have been due to the acquisition of TCam-2 cell adhesive ability to specific extracellular matrix substrates of basal lamina (laminin) or of the stromal interstitial compartment (fibronectin) [27]. As reported TCam-2 cells adhere more efficiently to fibronectin compared to other substrates, however the EW-induced TCam-2 cell relative adhesion increase seems to be equal regardless the substrates used. Consistent with these data we observed that EW did not modulate neither β1 integrin subunit (that forms both a laminin and a fibronectin receptor [27]) nor vinculin membrane cytoskeleton adaptor protein. These observations indicate that, even if EW was able to induce TCam-2 adhesion/spreading increase both on plastic or basal lamina and interstitial extracellular matrix substrates, it does not seem to trigger specific integrin/focal adhesion machinery at least for the adhesion molecules considered.

The analysis of actin distribution pattern allowed us to observe morphological and cytoskeleton modifications in EW treated cells in 24 to 72 hours of culture. These changes are characterized by a well-ordered cortical distribution of the actin filaments in correspondence to cell-cell contacts. Morphological changes via reorganization of adhesion molecules and actin fibres are important next steps in metastatic progression [31,32].

Quantitative morphological assessment enables us to ascertain the dramatic change in shape configuration of TCam-2, induced by EW. Indeed, cells exposed to EW assumed a more rounded profile, as documented by the reduced FD value and an increase in both Circularity and Solidity, highlighting how the cell acquires a less invasive phenotype [29].

Moreover, decrease in Fractal Dimension suggests that the morphological complexity is reduced after EW exposition. Overall, these data suggest a profound change in shape reorganization triggered by EW and reinforce the presented observations obtained in studying cell-to-cell relationships.

During tumour progression, transformed epithelial cells frequently lose E-cadherin-mediated adhesions. The E-cadherin loss in tumours can be mainly ascribed to inactivating mutations, epigenetic silencing, proteolytic cleavage, and proteosomal degradation [33]. In tumour cells, E-cadherin has shown properties of a tumour suppressor, as cells become more invasive when E-cadherin expression is down-regulated [34]. In human testis, E-cadherin is weakly expressed by PGC and foetal male germ cells, as well as by intratubular seminoma, whereas invasive seminoma does not express this particular junctional protein [35]. Interestingly, we demonstrated that EW was able to up-regulate E-cadherin, and the cadherin associated protein β-catenin, in TCam-2 cells. Immunofluorescence analysis revealed that both proteins were barely detectable in control samples whereas in EW administered cells strong cortical signals were observed in scattered groups of cells. According to these molecular data, TEM analysis revealed the presence of adherens junctions only in EW cultured cells. It is important to highlight that, previous TEM studies on TCam-2 cells demonstrated the complete absence of intercellular connection structures [22].

Gap junction loss is also related to seminoma neoplastic progression. Studies have defined that the transition from pre-invasive carcinoma in situ to seminoma is accompanied by a reduced expression in Sertoli cells and germ cells of the gap-junction related protein connexin-43 [36]. This protein represents the predominant testicular connexin, it was found in spermatogonia and spermatocytes and its down-regulation is also related with ipofertility [37-40]. Our data suggest that EW not only up-regulates connexin-43 in TCam-2 seminoma cells, but also completely modifies the cellular localization of that molecule. In particular we detected no connexin-43 membranous staining in the control samples while a strong perinuclear signal, compatible with a Golgi apparatus localization, was observed. On the contrary, EW-cultured cells showed a typical connexin-43 membranous staining at the level of cell-cell contacts. In accordance with our observation, data from related literature have shown that seminoma samples are characterized by an aberrant connexin-43 localization in the Golgi apparatus and the authors speculated that this delocalization may participate in tumour progression [41]. Consistent with these data, TEM analysis revealed the presence of structures compatible with gap junctional complexes only in EW treated samples. It is noteworthy that in a previous study we already showed that EW treatment was able to trigger the formation of junctional complexes which may address the hypothesis that the described differentiating effect is not restricted to seminoma cells [17].

Finally, we assessed whether EW might affect seminoma cells migratory capability. Cell migration plays a key role in metastatic dissemination of tumour cells from the primary tumour to local and distant sites [42-44]. Chemotaxis plays a central role in tumour progression such as metastatic invasion and dissemination, angiogenesis and immune cell extravasations [45]. We performed Boyden chamber assays to demonstrate that EW is able to inhibit TCam-2 cells chemo-attraction in response to low doses of serum. This inhibition is lost when chemotaxis is induced by high doses of serum, suggesting that the concentration of local growth factors is fundamental for the seminoma cell chemo-polarized migratory behaviour. Moreover we assessed whether E-cadherin and β-catenin, due to their up-regulation in response to EW administration, could have been involved in the TCam-2 reported migration differences. For this reason we performed Boyden chamber assays also on TCam-2 cells previously E-cadherin or β-catenin silenced and we observed a rescue of the migratory capacity of EW treated TCam-2 cells. This set of experiments allowed us to conclude that these two molecules are strongly involved in the modulation of EW-induced chemo-polarized migratory behaviour and their down-regulation could account of the metastatic potential of seminoma cells.

In addition to chemo-sensitive cell migration, we further studied TCam-2 collective cell migration by wound healing assay. This assay introduces a cell-free region where a physical barrier is applied before cell seeding. The removal of the physical barrier initiated the cell migration and this phenomenon was recorded by time lapse analysis. We found that EW treatment was able to significantly delay the wound closure, suggesting again its ability to modify cancer cell motility. Interestingly wound closure is not due to an increase of proliferating cells since the number of mitotic cells, counted at 24 and 48 hours of culture during wound healing assay does not change in EW versus control condition. On the basis of these data we can conclude that the observed delay in wound closure can be explained only by an acquisition of different cell dynamism in response to EW administration.

All together these “in vitro” observations allowed us to hypothesize that EW treatment could rescue, at least in part, the metastatic behaviour of seminoma cells.
