# Correction: TCam-2 Seminoma Cells Exposed to Egg-Derived Microenvironment Modify Their Shape, Adhesive Pattern and Migratory Behaviour: A Molecular and Morphometric Analysis

**DOI:** 10.1371/annotation/1c70ff15-389c-4ad7-b333-6615e98713d0

**Published:** 2013-10-03

**Authors:** Francesca Ferranti, Fabrizio D’Anselmi, Maria Caruso, Vittorio Lei, Simona Dinicola, Alessia Pasqualato, Alessandra Cucina, Alessandro Palombo, Giulia Ricci, Angela Catizone, Mariano Bizzarri

The first two authors, Francesca Ferranti and Fabrizio D’Anselmi should be noted as contributing equally. 

